# Comparative cohort study of Duhamel and endorectal pull-through for Hirschsprung’s disease

**DOI:** 10.1093/bjsopen/zrab143

**Published:** 2022-02-02

**Authors:** Joseph R. Davidson, Annika Mutanen, Malla Salli, Kristiina Kyrklund, Paolo De Coppi, Joe Curry, Simon Eaton, Mikko P. Pakarinen

**Affiliations:** 1 Department of Specialist Neonatal and Paediatric Surgery, Great Ormond Street Hospital for Children, London, UK; 2 Stem Cells and Regenerative Medicine Section, GOS-UCL Institute of Child Health, London, UK; 3 Department of Pediatric Surgery, Helsinki University Hospital and New Children’s Hospital, Helsinki, Finland

## Abstract

**Background:**

There are limited data available to compare outcomes between surgical approaches for Hirschsprung’s disease. Duhamel and endorectal pull-through (ERPT) are two of the most common procedures performed worldwide.

**Methods:**

Objective outcomes were compared between contemporary cohorts (aged 4–32 years) after Duhamel or ERPT using case–control methodology. Data were collected using prospectively administered standardized questionnaires on bowel and bladder function and quality of life (Pediatric Quality of Life Inventory, Short form 36 and Gastrointestinal Quality of Life Index). Patients were compared in two age groups (18 years and younger and older than 18 years) and reference made to normative control data. Multivariable analysis explored factors associated with poor outcomes.

**Results:**

Cohorts were well matched by demographics, disease characteristics and incidence of postoperative complications (120 patients who underwent Duhamel *versus* 57 patients who had ERPT). Bowel function scores were similar between groups. Patients who underwent Duhamel demonstrated worse constipation and inferior faecal awareness scores (*P* < 0.01 for both age groups). Recurrent postoperative enterocolitis was significantly more common after ERPT (34 *versus* 6 per cent; odds ratio 15.56 (95 per cent c.i. 6.19 to 39.24; *P* < 0.0001)). On multivariable analysis, poor bowel outcome was the only factor significantly associated with poor urinary outcome (adjusted odds ratio 6.66 (95 per cent c.i. 1.74 to 25.50; *P* = 0.006)) and was significantly associated with markedly reduced quality of life (QoL) in all instruments used (*P* < 0.001 for all). There were no associations between QoL measures and pull-through technique.

**Conclusion:**

Outcomes from Duhamel and ERPT are good in the majority of cases, with comparable bowel function scores. Constipation and impaired faecal awareness were more prevalent after Duhamel, with differences sustained in adulthood. Recurrent enterocolitis was significantly more prevalent after ERPT. Clustering of poor QoL and poor functional outcomes were observed in both cohorts, with seemingly little effect by choice of surgical procedure in terms of QoL.

## Teaser

This study compares outcomes in two contemporary cohorts with Hirschsprung’s who have undergone differing surgical reconstruction. Patients after Duhamel are affected by worse constipation and appear to have reduced rectal sensation than those who have undergone endorectal pull-through, who themselves appear to be at higher risk of postoperative Hirschsprung’s-associated enterocolitis. Quality of life is good in the majority of patients but does seem to be highly correlated with poor functional outcome, demonstrating a need to support these patients with long-term multidisciplinary support.

## Introduction

**Fig. 1:**
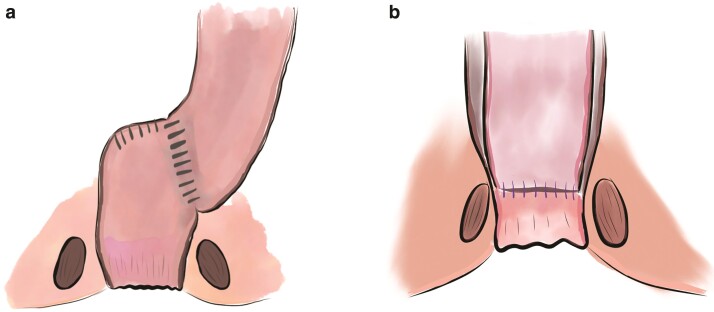
Illustrations of the surgical procedures **a** The Duhamel procedure involves a transabdominal retrorectal dissection and pull-through of ganglionic bowel to the upper limit of the internal anal sphincter, with a stapled anastomosis leaving a residual anterior rectum of Hirschsprung’s-affected bowel. **b** The endorectal pull-through procedure involves transanal circumferential dissection in the submucosal plane, creating a seromuscular cuff through which ganglionic bowel is pulled and a sutured coloanal anastomosis formed.

The principle of definitive surgical management of Hirschsprung’s disease (HSCR) involves the anastomosis of ganglionic bowel at, or close to, the anal sphincter to allow continent expulsion of stool. It is now widely accepted that the remaining ganglionic intestine may be somewhat abnormal in terms of mucosal function and motility, however most patients can achieve adequate bowel function following surgery^1^. Although a variety of techniques and modifications have evolved for the pull-through procedure, the Duhamel (*Fig. 1a*) and endorectal pull-through (ERPT) (*Fig. 1b*) operations remain the most widely adopted for HSCR worldwide.

The Duhamel pull-through, named after Bernard Duhamel, involves a longitudinal, side-to-side anastomosis of the native aganglionic rectum with recruited ganglionic proximal colon^2^. The justification for leaving the distal aganglionic rectum *in situ* is to retain a faecal reservoir and to avoid circumferential rectal dissection, which can cause iatrogenic injury to pelvic nerves and urogenital structures. Postoperative complications commonly reported are residual constipation, sometimes related to spur formation at the anastomosis as well as distension of the residual rectum—or Duhamel pouch.

The ERPT was initially published by Franco Soave of Genova^3^, and involves a submucosal dissection for several centimetres, resulting in a muscular cuff of native rectum. Modifications by De La Torre and Langer have since been widely adopted such that the procedure can now be performed through a transanal approach^4,5^. It has been suggested that a totally transanal ERPT carries a higher risk of iatrogenic sphincter injury through prolonged stretching of the anal sphincter^6^, but may reduce injury to perirectal structures low in the pelvis. A totally transanal approach is feasible for rectosigmoid (short/standard) segment disease but many centres currently opt for colonic mobilization by either laparoscopy or laparotomy to avoid prolonged anal sphincter retraction and to visualize and prevent abnormal twisting of the pulled-through bowel.

Observational series have reported discrepant results between different surgical approaches in terms of bowel function and postoperative complications such as Hirschsprung’s-associated enterocolitis (HAEC)^7^. Direct comparison has been challenging due to variability in outcome assessment with various scoring systems and self-designed criteria being applied to data retrospectively. Series that have directly compared outcomes with the same criteria have been limited in being single-centre^8,9^. The authors’ aim was to compare Duhamel and ERPT with a cross-sectional assessment of outcomes in relation to matched normal population controls in contemporaneous, age-matched cohorts of patients with HSCR at two large-volume referral centres.

## Methods

### Study design

Patients with HSCR treated either at Helsinki University Hospital (HUH; 1987–2011), Finland, or Great Ormond Street Hospital (GOSH; 1977–2013), London, UK were eligible for inclusion. Both centres are regional referral centres for paediatric colorectal surgery and patients undergoing Duhamel (GOSH) or ERPT (HUH) were included. This study was designed with case–control methodology, comparing patients with Duhamel and ERPT across a number of domains and was reported according to the STROBE statement^10^. These groups were extracted from larger study cohorts of outcome data which have previously been published^11,12^, hence this was novel analysis of published cohorts answering a distinct question with clinical relevance—whether there is a measurable difference in the functional or quality-of-life outcomes between ERPT or Duhamel. Outcomes were compared with national reference data as available.

### Technical aspects of procedures

The technical aspects of the Duhamel procedures performed at GOSH have been consistent throughout the study period; the technique was published by Kiely in 2005^13^. It includes a low anastomosis (inferior margin at the upper limit of the internal anal sphincter) and relatively short residual rectum. Current use of laparotomy *versus* laparoscopy is defined by surgeon preference.

ERPT has been the procedure of choice at HUH since 1987 and has been consistent throughout the study period and completed by the same team of paediatric colorectal surgeons or surgeons trained by them^11^. ERPT is performed either completely transanally or in combination with transabdominal colonic mobilization by mini-laparotomy or laparoscopy, defined by surgeon preference. It includes a transanal mucosectomy approximately 5 mm above the dentate line for distance of 3–4 cm, proceeding to full-thickness dissection thereafter. Patients with TCA are managed with restorative proctocolectomy and short J-pouch ileoanal anastomosis and protective temporary ileostomy^14,15^.

### Ethics

Local and national ethical approvals were granted in both countries: (GOSH 17DS04; UK NHS REC 17/LO/1692; HUH TMK03§261). Written informed consent to participate in the study was obtained from patients and/or their guardians.

### Inclusion and exclusion criteria

The cohort of eligible patients who had undergone ERPT had an age range of 4–32 years and patients of the same age range from the Duhamel cohort were included for comparison. Patients with other pull-through techniques were excluded, as were patients who underwent primary surgery elsewhere. Patients with syndromic association or considerable learning disability were excluded as the authors have demonstrated previously these patients may have variable outcomes for reasons other than HSCR and its surgical management^16^.

### Data collected

Clinical case data were retrieved from patients’ medical records, including demographic, surgical and disease-specific data. Post-surgical complications were assessed according to Clavian-Dindo^17^. HSCR segment length was defined using standard definitions: rectosigmoid, long (extending past the sigmoid colon) and total colonic aganglionosis (TCA)^18^. Long-segment disease and TCA were pooled together as ‘extended segment’ for the purposes of their use as a variable in multivariable analysis.

Episodes of HAEC were defined by the clinician on an intention-to-treat basis; patients were also asked to report any historic episodes of HAEC and recurrent HAEC, defined as more than four episodes in a year.

Patients were contacted by researchers who were not directly involved in their surgical or medical care (J.D., M.S.). Patients and their families were invited to complete questionnaires with a number of outcomes assessed.

Functional outcomes were measured using the Bowel Function Score (BFS) described by Rintala and Lindahl (maximum, 20: good, 17 or greater; moderate, greater than 12; poor, 12 or less)^19^. Patients requiring antegrade continence enemas (ACE) or enterostomy to control bowel function were not able to be assessed by the questionnaire and were deemed to have poorer quality-of-life outcome as per previous studies^12,20^.

Urinary symptoms were explored using a modified Danish Prostatic Symptom Score^21^, which has been used previously in studies of children with HSCR^22^. These were compared with reference outcomes from the Finnish general population; and as age-related changes in bowel and bladder function occur through childhood^23,24^, the authors compared those patients under 18 years and those 18 years of age and above separately.

Symptom-specific quality of life was assessed with the Gastrointestinal Quality of Life Index (GIQLI^©^) in patients at least 18 years of age^25^, where the cut-off value for a normal outcome is 105 out of 144. Overall GIQLI^©^ score and subdomain scores were also compared with outcomes from age-matched, pooled control groups from several previously published studies^11,26,27^.

General quality-of-life instruments, Pediatric Quality of Life Inventory (PedsQL™) and Short form 36 (SF-36), were also used to assess outcomes in children and adults respectively. These are known to vary significantly between different national populations. As such, PedsQL™ scores were compared with normative data from the UK^28^ or Finland (unpublished, collected by the authors). Patient-derived outcomes were standardized to normative data using a Z-score transformation allowing comparison between countries and allowing a valid comparative analysis of procedures. SF-36 data were analysed with respect to normative data from the UK^29^ or Finland^30^ (pooled with additional unpublished data collected by the authors). Physical and mental component scores were calculated using domain weightings as described by Taft^31^. Briefly, each patient’s score is normalized against a gender- and age-matched population mean and standard deviation; calculation of these component scores allows for meaningful comparison of cohorts and improves statistical power by limiting the number of comparisons to two (as opposed to comparing all eight domains of the SF-36). Examination of quality-of-life metrics in such a way allows for a measure against an age- and gender-matched score from the same country—thus providing a more reliable impression of quality-of-life impact.

### Statistical analysis

Baseline and disease-specific data were compared using Fisher’s exact test for categorical data and Mann–Whitney *U* test for continuous variables. Kruskal–Wallis with Dunn’s test for multiple comparisons was used to compare outcomes between patient groups and controls for bowel and urological function, and GIQLI^©^; here, the use of non-parametric testing allowed for appropriate comparison of the ordinal data generated from such scoring systems. Where multiple domains were compared (within BFS or GIQLI^©^), *P* values were further corrected by Bonferroni’s method to reduce the risk of type I error. Where differences between scores were statistically different, the authors opted to utilize a minimum clinically important difference; this can be calculated based upon distributions of the two cohorts with Cohen’s *d* with a cut-off value of 0.5. For non-parametric testing, η^2^ was converted to Cohen’s *d* as per the transformation available at https://www.psychometrica.de/effect_size.html. Multivariable analyses were performed in order to identify factors associated with poor outcome (variables available along with results in *Supplementary material*).

## Results

### Demographics and operative management

Sixty-three per cent of invited patients managed with ERPT and 64 per cent of patients undergoing Duhamel participated, with no missing data points (*Table 1*). Operative approaches differed between cohorts in both the younger and older groups: use of preoperative defunctioning stoma was considerably higher in patients undergoing Duhamel in both age groups, whereas the patients undergoing ERPT were considerably younger at the time of their pull-through in the modern era. Use of open surgery was similar, with the vast majority of patients 18 years or older having undergone an open approach, and approximately two thirds in the less than 18 years group.

**Table 1:** Demographics, disease characteristics and operative management

	Under 18 years		*P*†	Adults		*P*†
	Duhamel (*n* = 45)	ERPT (*n* = 39)		Duhamel (*n* = 75)	ERPT (*n* = 18)	
**Demographics**						
Age (years)*	11 (5–18)	10 (4–18)	0.875	26 (19–32)	23 (19–32)	**0.034**
Male : female	35 : 10	31 : 8	1.000	57 : 18	11 : 7	0.240
**Disease characteristics**						
Family history	7	9	0.416	11	4	0.479
Rectosigmoid	36	32	0.840	58	17	0.302
Long	5	5		11	1	
TCA	4	2		6	0	
**Operative management**						
Laparotomy	31	24	0.500	73	16	0.167
Laparoscopy assisted	14	2		2	1	
Totally transanal	–	13		–	1	
Preoperative stoma	21	3	**<0.0001**	47	3	**0.0005**
Age at pull-through (days)*	121 (47–2973)	36 (11–3525)	**0.001**	152 (7–1879)	125 (21–3316)	0.865

Values in parentheses are percentages unless stated otherwise; *values are median (range). Bold values are statistical significance (i.e. *P* < 0.05). †Mann–Whitney *U,* Fisher’s exact, or Chi-squared for trend tests. ERPT, endorectal pull-through; TCA, total colonic aganglionosis.

**Fig. 2:**
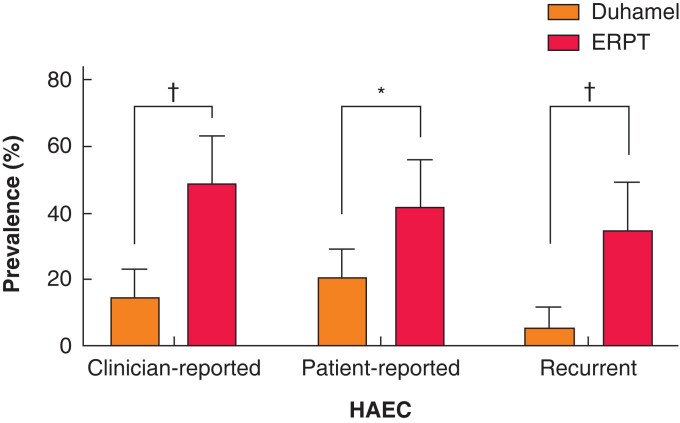
Incidence of Hirschsprung’s-associated enterocolitis according to clinical records and patient-reported episodes Error bars represent 95 per cent confidence interval of proportion. *P < 0.010, †*P* < 0.001 (Fisher’s exact test). HAEC, Hirschsprung’s-associated enterocolitis; ERPT, endorectal pull-through.

Postoperative complications are summarized in *Table 2*. The overall requirement for unplanned major surgery (including bowel obstruction, leak, stoma formation, revision surgery) was 13 per cent in Duhamel operations and 12 per cent in ERPT. As spur division in Duhamel and intrasphincteric botulinum toxin injection (both Duhamel and ERPT) required general anaesthesia, the overall complications Clavien–Dindo of grade IIIb and above were 27.5 and 19.3 per cent respectively (*P* = 0.27). Redo pull-through was required in six patients post-Duhamel and one patient post-ERPT. A significantly higher incidence of HAEC was observed after ERPT (49 *versus* 15 per cent after Duhamel for clinician-reported episodes; 42 *versus* 21 per cent for patient-reported episodes, 34 *versus* 6 per cent for recurrent HAEC (4 or more episodes in one year); *P* < 0.01 for all) (*Fig. 2*). All three definitions of HAEC were reported more in patients of a younger age, independent of the surgery they had undergone when this was included in multivariable analysis (*P* < 0.05 for all). When surgical procedure was explored with this analysis it continued to be a significant relationship for clinician-reported HAEC (adjusted odds ratio 4.35 (95 per cent c.i. 2.06 to 9.17; *P* < 0.001)) and patient- or clinician-reported recurrent HAEC (adjusted odds ratio 6.83 (95 per cent c.i. 2.61 to 17.85; *P* < 0.001)). It was not an independently significant factor in patient-reported isolated episodes (adjusted odds ratio 1.94 (95 per cent c.i. 0.94 to 4.03; *P* = 0.075)).

**Table 2:** Operative complications

	Duhamel (*n* = 120)	ERPT (*n* = 57)	*P* *
**Complications (Clavien-Dindo ≥IIIb)**	33	11	0.269
**Early**			
Leak	5	1	0.666
**Late**			
Stricture	0	0	–
Spur	16	–	–
Need for botulinum toxin	6	6	0.205
Unplanned major surgery	15	7	1.000
Postoperative stoma formation	7	1	0.280
Redo PT	6	1	0.431
ACE formed	4	2	1.000
**Current status**			
Stoma	3	0	0.552
ACE	0	2	0.102

Values in parentheses are percentages. *Fisher’s exact test. ERPT, endorectal pull-through; PT, pull-through; ACE, antegrade continence enemas.

### Bowel function outcomes

**Fig. 3:**
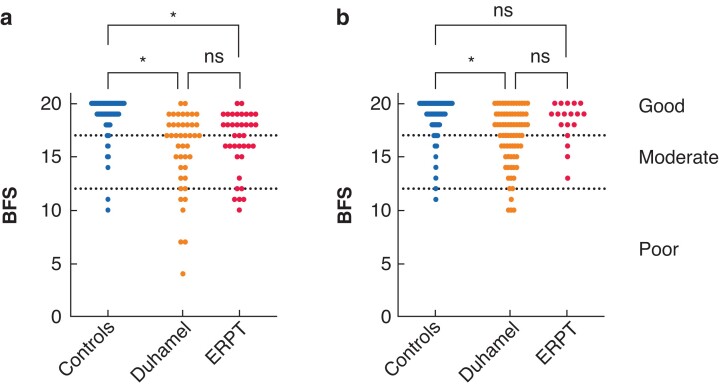
Overall Bowel Function Scores **a** Patients younger than 18 years. **b** Patients 18 years or older. *P < 0.0001 (Kruskal–Wallis and Dunn’s multiple comparisons test). BFS, Bowel Function Score; ERPT, endorectal pull-through; ns, not significant.

Overall BFS in patients less than 18 years was impaired relative to controls in both groups, with no difference between cohorts (Duhamel *versus* ERPT *versus* control; median (i.q.r.) 17 (14–18) *versus* 17 (15.5–19) *versus* 20 (19–20), overall Kruskal–Wallis *P* < 0.0001; *Fig. 3a*). The differences compared with controls were significant in both Duhamel (*d *= 0.84) and ERPT (*d *= 0.72). Patients 18 years and over with Duhamel also had impaired bowel function relative to controls (*P* < 0.0001; *d *= 0.81), however outcomes in patients who underwent ERPT were not statistically different from those in controls (median (i.q.r.) 18 (15–19) *versus* 19 (17.75–20) *versus* 19 (19–20), Kruskal–Wallis *P* < 0.0001, *Fig. 3b*). Combining both age groups, the number of patients with a poor outcome (score less than 12 or with stoma or ACE) was similar in both patient groups but worse than in controls (Duhamel *versus* ERPT *versus* control; 10 of 120 (8 per cent) *versus* 3 of 57 (5 per cent) (*P* = 0.5), *versus* 1 of 683 (0.1 per cent); *P* < 0.005 for both groups).

**Fig. 4:**
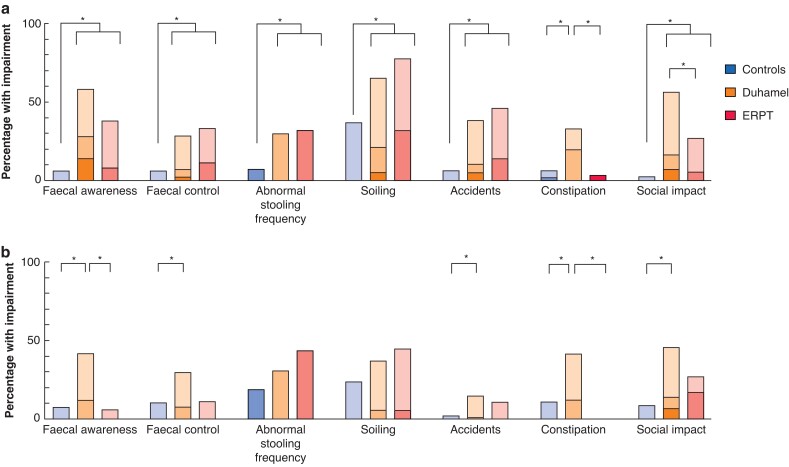
Domains of Bowel Function Scores in controls, and patients undergoing Duhamel and endorectal pull-through **a** Patients under 18 years. **b** Patients 18 years and older. *P < 0.001(score treated as ordinal data and compared with Kruskal–Wallis and Dunn’s test; *P* value further corrected for multiple comparisons (seven domains)). ERPT, endorectal pull-through. Severe/Frequent symptoms in darker shade with Milder/Infrequent symptoms in paler shade.

In patients under 18 years, individual bowel function domains were inferior to those of controls in all except constipation where only patients with Duhamel had inferior scores (*Fig. 4a*). Comparing ERPT and Duhamel in this younger group, patients with Duhamel had reported issues more frequently for constipation and social issues related to bowel function (*P* < 0.001). Severe issues in any domain were only reported by a minority of patients, with the most common domain being that of faecal awareness (no awareness at all in 6 of 45 Duhamel patients) *versus* 0 of 39 patients with ERPT). The only domain with severe issues reported in ERPT patients was constipation (requiring enemas in 1 patient *versus* no patients with Duhamel).

Considering BFS domains in adult patients (*Fig. 4b*), patients with Duhamel more frequently had issues with constipation and faecal awareness than those with ERPT or normal controls, there was also a higher incidence of issues withholding stool and social issues related to bowel function but compared with controls only. Severe issues were not reported by ERPT patients in any domain, and only in social impact by patients with Duhamel (5 patients, 7 per cent).

### Urinary symptoms

**Fig. 5:**
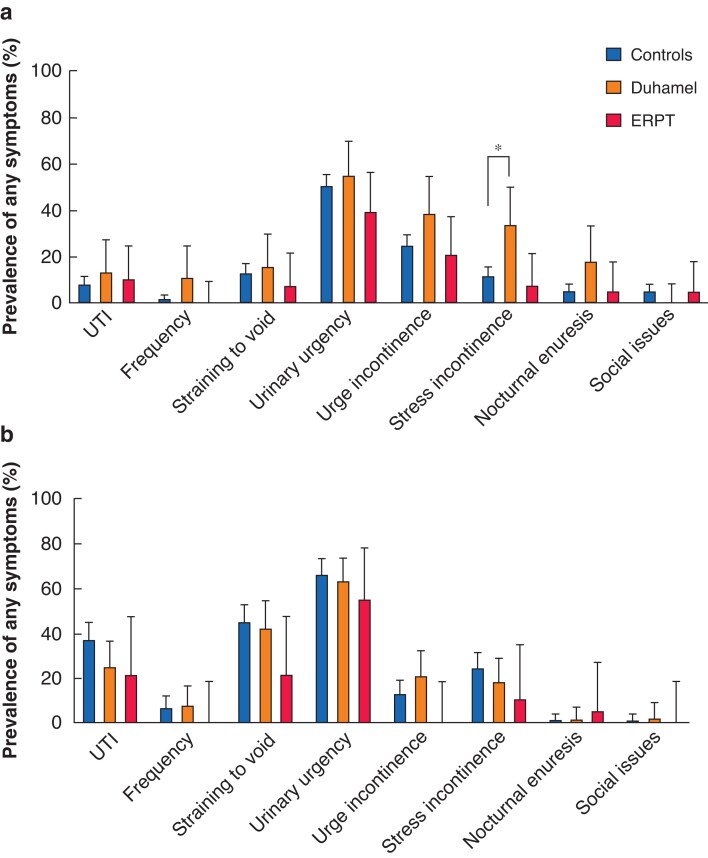
Urinary symptoms in controls, and patients undergoing Duhamel and endorectal pull-through **a** Patients under 18 years. **b** Patients 18 years and older. Error bars represent 95 per cent confidence interval of proportion. *P = 0.007 (Fisher’s exact test). ERPT, endorectal pull-through; UTI, urinary tract infection.

Weekly or daily continence issues were infrequent across all patients: urge incontinence, six of 175 (3 per cent); stress incontinence, four of 175 (2 per cent); nocturnal enuresis, four of 175 (2 per cent) and social issues related to urinary incontinence were reported in only four of 175 (2 per cent). Low frequency (less often than weekly) symptoms were assessed in comparison between cohorts and compared with controls (*Fig. 5*). Incidence of previous urinary tract infection was similar between patient groups and controls. Among patients under 18 years, Duhamel patients had a significantly higher incidence of any stress incontinence symptoms than controls (corrected *P* = 0.007), however symptoms were less than weekly frequency in all but one of 44 patients. Adult patients compared with controls had no differences in urinary symptoms or incontinence after Bonferroni correction for comparisons.

### Quality-of-life outcomes

**Fig. 6:**
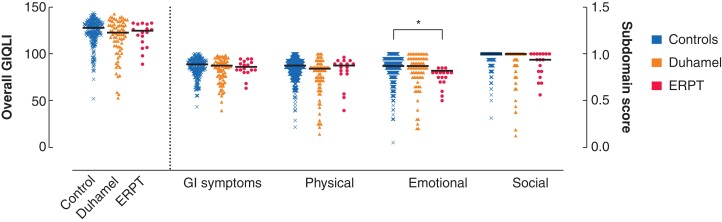
Gastrointestinal Quality of Life Index scores for controls, and patients undergoing Duhamel and endorectal pull-through Overall score maximum 144. Subdomain scores calculated as a proportion of a theoretical maximum (scaled to a proportion of 1). Median value given as black horizontal line. *P = 0.008 (corrected *P* value, Kruskal–Wallis and Dunn’s test). ERPT, endorectal pull-through; GI, gastrointestinal; GIQLI, Gastrointestinal Quality of Life Index.

Overall GIQLI^©^ scores (adults) were comparable between patients and controls (*Fig. 6*), with the only subdomain difference being a slightly lower emotional score for ERPT patients relative to controls (*d *= 0.47; *P* = 0.008) but not Duhamel patients (*d *= 0.54; *P* = 0.054). Specifically, the GIQLI^©^ asks ‘To what extent has your sexual life been impacted because of your illness?’; there was no difference in the responses by procedure (Mann–Whitney *U* test; *P* = 0.337). However, when the authors explored responses by sex, they found scores in females to be significantly lower than in males (*P* = 0.003; *d* = 0.51).

**Fig. 7:**
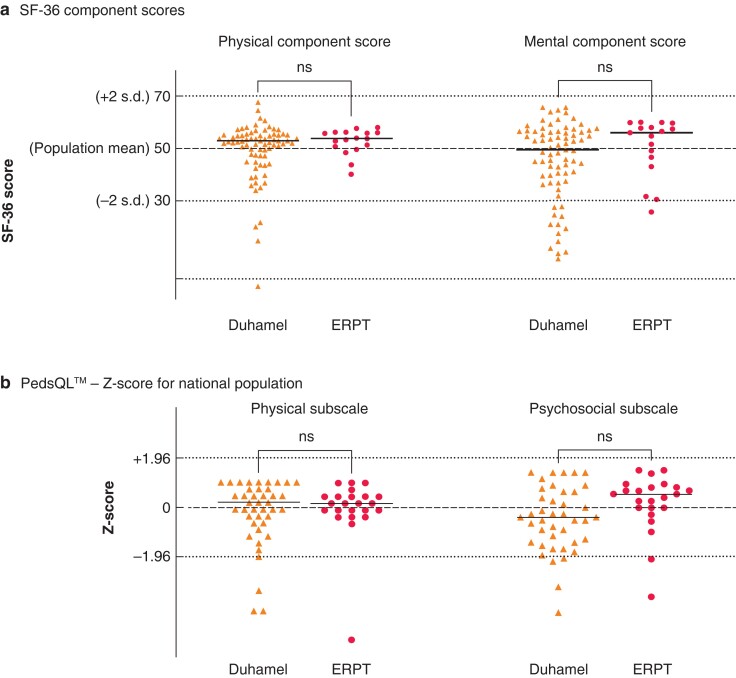
General quality-of-life scores normalized for country, age and gender in patients after Duhamel and endorectal pull-through **a** Short form 36 (SF-36) in adult patients. **b** Pediatric Quality of Life Inventory (PedsQL™) in patients less than 18 years. Comparison with unpaired Welch’s t-test with Bonferroni correction for dual comparison. ERPT, endorectal pull-through; ns, not significant. Horizontal lines on each data series represents the mean value.

General quality-of-life indices were explored in both adults (SF-36) and children (PedsQL™). Both scores were normalized to age- and gender-matched general population data from the respective countries^29,30^; this created physical component scores (PCSs) and mental component scores (MCSs) for SF-36 (*Fig. 7a*). Neither score was significantly different between Duhamel or ERPT patients, although more Duhamel patients had a score below 2 standard deviations of normal (component score below 30) for both PCS (4 of 75 *versus* 0 of 18) and MCS (13 of 75 *versus* 1 of 18).

PedsQL™ scores in patients were normalized to age-matched controls to produce Z-scores; physical and psychosocial domains were analysed separately (*Fig. 7b*). Again, there were no statistical differences between the scores in either subscale, with the statistical difference on the psychosocial scale becoming non-significant after correction despite being considered above the threshold for a minimum clinically significant difference (*P* = 0.099; *d *= 0.533).

### Determinants of poor outcome

The data were also analysed using multivariable binary and logistic models (*Supplementary material*). Poor bowel outcome, defined as a BFS less than 12 or need for ACE or stoma, was associated only with age less than 18 years at the time of study (adjusted odds ratio 3.92 (95 per cent c.i. 1.30 to 11.83; *P* = 0.016)) and weekly/daily urinary incontinence was associated only with the previously defined poor bowel outcome (adjusted odds ratio 6.66 (95 per cent c.i. 1.74 to 25.50; *P* = 0.006)).

GIQLI^©^ scores were analysed with multivariable linear regression and lower scores (maximum 144; Beta + 95 per cent c.i.) were seen in patients with poor bowel outcome (41.29 (26.48–56.11); *P* < 0.001), extended segment Hirschsprung’s (14.19 (5.49–22.88); *P* = 0.002) and female sex (7.79 (0.06–15.52); *P* = 0.048).

SF-36 component scores (mean(s.d.) 50(10)) were significantly lower in patients with poor bowel outcome (PCS reduced by 16.53 (7.59–25.47); MCS by 24.27 (11.86–36.69); *P* < 0.001 for both). Similarly, the PedsQL™ overall Z-scores were reduced markedly in patients with poor bowel outcome (reduced by 2.30 (1.45–3.14); *P* < 0.001). The type of surgical procedure had no association with quality-of-life outcomes in any of these models (*P* = 0.38–0.79).

## Discussion

This study compares outcomes from two well matched cohorts of HSCR patients from two institutions undergoing either an ERPT or Duhamel procedure with no difference in major postoperative complications. However, the authors found a higher incidence of HAEC after ERPT and more frequent issues with faecal awareness and constipation after Duhamel. The significantly higher incidence of HAEC in patients undergoing ERPT is greater than reported in a meta-analysis of patients following this procedure^32^. However, HAEC may also be underdiagnosed, and rates were over 50 per cent after pull-through according to high-sensitivity criteria in a multi-institution series where the provider-defined rate was only 28 per cent^33^. Taking a pragmatic approach to retrospectively collected data, the authors opted to use an intention-to-treat definition for HAEC. Given this, and the supplementation of this information with patient recall, it is possible that the observed differences might be, in part, related to differing thresholds for treatment and variable documentation. HAEC was more common in younger patients and, although this may represent lower thresholds for case definition, it suggests an element of recall bias and the limitation of retrospective studies. The differences between the cohorts for clinician-reported, patient-reported, and recurrent HAEC suggest the difference between ERPT and Duhamel is real. Historic patient cohorts from Finland with Duhamel pull-through^34^ (preceding the ERPT cohort) demonstrated HAEC incidence similar to that in this study. The authors hypothesize that HAEC may be more common after ERPT due to some restriction from the seromuscular cuff, or perhaps in post-Duhamel anatomy, the residual anterior rectum (Duhamel pouch) may allow for a degree of distension during obstructive stooling due to external sphincter achalasia.

The noted specific differences in the bowel-function profile of patients are in keeping with the published literature^8,9^. Differences in faecal awareness were also apparent in adult patients, with reduced awareness reported more commonly in patients after Duhamel. The reasons underlying this are unclear however; the authors propose that enduring constipation and rectal distension may interfere with faecal awareness in some patients.

Urological outcomes have not been well reported in the literature, however in the authors’ comparative analysis, symptoms did not differ significantly between patients by procedure, with very few patients reporting frequent incontinence episodes. Urinary incontinence symptoms were strongly associated with a poor bowel outcome. This confirms data from the authors’ single-centre analysis including patients with learning disability, demonstrating urinary incontinence as the single most predictive factor of frequent incontinence episodes^16^. This study benefits from comparison with age-matched controls for functional scores^23,24^. Sexual function and fertility data for patients with HSCR have not been well defined; the authors’ own single-centre data suggest that adult women appear to more frequently be affected by sexual dysfunction and potential subfertility after Duhamel surgery in infancy^35^. However, with limited numbers from a single-centre study, and a lack of comparative group who have undergone alternative surgical reconstruction, this warrants further research.

The quality-of-life outcomes described here are reassuring and provide important data to emphasize further the intrinsic link with functional outcome, with poor bowel outcome being uniformly associated with poor quality-of-life score in any metric used. Lower GIQLI^©^ scores were independently associated with extended segment disease, which is a known predictor also of poorer functional outcome. Within this study group it is likely that the numbers of longer segment patients were not sufficient to demonstrate the same effect on multivariable analysis. Using age- and sex-matched controlled outcomes to normalize quality-of-life data is helpful to demonstrate effects of a condition on the outcome of interest^36^. Controlling for quality-of-life outcomes with country-specific data also helps avoid any variation in results that may be due to nuances of language affecting the cross-cultural validity of an instrument^37^. The lack of UK-based control data for the functional instruments presented here is a potential source of bias of the results; it is important to note that there is a limitation in the validity of questionnaires to assess bowel function in HSCR patients, and that none have been validated in the Finnish language. Furthermore, although the response rates of 63 and 64 per cent are relatively high for cross-sectional outcome collection of cohorts treated over several decades, this proportion of responses maintains a possibility of reporting bias. The previously published larger cohorts of the two centres reporting data here have not shown any significant differences between included patients and non-respondents on dropout analysis^11,12^.

The data presented in this study demonstrate that there are surgery-specific outcomes that should be considered when counselling families and consenting for surgery. Although the quality-of-life outcomes in either surgical approach appear to be within normal limits, there is a population of patients with HSCR who struggle with bowel function and suffer consequent significant impact on their longer-term quality of life. This reinforces a need to provide multidisciplinary support in those identified to be at risk of a poor outcome as they transition out of paediatric services. While current management of HSCR is able to deliver a satisfactory outcome in most, reconstructive surgery does not cure the disease. Further research is needed to study genetic and epigenetic aspects of this condition^38^, developmental biology^39^ and immunological changes in HSCR^40,41^ to deliver the best outlook for these patients.

## Supplementary Material

zrab143_Supplementary_Data
