# Supplementary material for: Comparative cohort study of Duhamel and endorectal pull-through for Hirschsprung’s disease
Source: BJS Open. 2022 Feb 2;6(1):zrab143. doi: 10.1093/bjsopen/zrab143 (PMC8830754; doi:10.1093/bjsopen/zrab143)
Supplement: zrab143_Supplementary_Data [file zrab143_supplementary_data.zip › Supplementary_material.docx]

**Regression Tables – For Online Supplement**

Determinants of Poor Bowel Outcome (Logistic Regression, BFS<12 / Current Use of Stoma or ACE)

|  | OR | aOR | p |
| --- | --- | --- | --- |
| Female Sex | 0.81 [0.26-2.56] | 0.91 [0.28 – 3.03] | 0.879 |
| Extended Segment | 1.30 [0.36-4.75] | 1.33 [ 0.36 – 5.00] | 0.670 |
| **Age at Study <18y** | **3.52 [1.21-10.24]** | **3.92 [1.30 – 11.83]** | **0.016** |
| Duhamel | 1.03 [0.37-2.87] | 1.54 [0.53 – 4.50] | 0.456 |

Determinants of Poor Urinary Outcome (Logistic Regression, Weekly/Daily Incontinence)

|  | OR | aOR | p |
| --- | --- | --- | --- |
| Female Sex | 1.83 [0.58 – 5.78] | 1.84 [0.53 – 6.44] | 0.334 |
| Extended Segment | 2.57 [0.80-8.23] | 2.72 [0.78 – 9.54] | 0.118 |
| Age at Study <18y | 0.89 [0.30-2.70] | 0.93 [0.27 – 3.23] | 0.902 |
| Duhamel | 3.06 [0.66-14.1] | 3.03 [0.59 – 15.54] | 0.182 |
| **Poor Bowel Outcome** | **5.91 [1.74-20.08]** | **6.66 [1.74 – 25.50]** | **0.006** |

Correlates with QOL (Multivariate Linear Regression)

| OVERALL PEDQL Z-SCORE | Mean Diff. | B [95% C.I.] | p |
| --- | --- | --- | --- |
| Female Sex | 0.06 [-0.63 – 0.76] | 0.23 [-0.51 – 0.98] | 0.534 |
| Extended Segment | 0.25 [-0.52 – 1.01] | 0.01 [-0.68 – 0.69] | 0.985 |
| Duhamel | -0.61 [-1.29 – 0.07] | -0.26 [-0.83 – 0.32] | 0.379 |
| **Poor Bowel Outcome** | **-2.44 [-3.62 - -1.26]** | **-2.30 [-3.14 – -1.45]** | **<0.001** |
| Poor Urinary Outcome | -1.23 [-3.24 – 0.78] | -0.31 [-1.30 – 0.69] | 0.540 |

| OVERALL GIQLI (/144) | Mean Diff. | B [95% C.I.] | p |
| --- | --- | --- | --- |
| **Female Sex** | **-7.56 [-16.8 – 1.67]** | **-7.79 [-15.52 – -0.06]** | **0.048** |
| **Extended Segment** | **-13.28 [-25.81 – -0.75]** | **-14.19 [-22.88 – -5.49]** | **0.002** |
| Duhamel | -3.25 [-11.37 – 4.86] | -1.19 [-10.03 – 7.66] | 0.790 |
| **Poor Bowel Outcome** | **-43.09 [-82.11 - -4.08]** | **-41.29 [-56.11 – -26.48]** | **<0.001** |
| Poor Urinary Outcome | -15.53 [-44.99 – 13.82] | -6.91 [-19.86 – 6.03] | 0.291 |

| SF36 Phys CS | Mean Diff. | B [95% C.I.] | p |
| --- | --- | --- | --- |
| Female Sex | 1.43 [-3.14 – 6.00] | 1.69 [-2.97 – 6.36] | 0.473 |
| Extended Segment | -3.41 [-9.12 – 2.31] | -2.46 [-7.71 – 2.79] | 0.354 |
| Duhamel | -3.26 [-6.76 – 0.24] | -1.12 [-6.45 – 4.22] | 0.679 |
| **Poor Bowel Outcome** | **-17.00 [-38.13 – 4.12]** | **-16.53 [-25.47 – -7.59]** | **<0.001** |
| Poor Urinary Outcome | -5.76 [-19.14 – 7.62] | -3,62 [-11.43 – 4.19] | 0.359 |

| SF36 Ment CS | Mean Diff. | B [95% C.I.] | p |
| --- | --- | --- | --- |
| Female Sex | -4.30 [-11.15 – 2.54] | -4.19 [-10.67 – 2.29] | 0.202 |
| Extended Segment | -2.24 [-11.69 – 7.22] | -2.29 [-9.58 – 5.00] | 0.534 |
| Duhamel | -5.01 [-11.63 – 1.61] | -3.58 [-11.00 – 3.83] | 0.339 |
| **Poor Bowel Outcome** | **-25.75 [-44.19 – -7.32]** | **-24.27 [-36.69 – -11.86]** | **<0.001** |
| Poor Urinary Outcome | -5.87 [-21.49 – 9.75] | -1.54 [-12.39 – 9.31] | 0.778 |
